# Examination of blood filtration membrane removal ability of HMGB1

**DOI:** 10.1186/cc11697

**Published:** 2012-11-14

**Authors:** H Imahase, Y Sakamoto, M Kusunose, H Koami, Y Nishimura, A Goto, T Yamashita, A Nakashima, T Iwamura, N Kutsukata

**Affiliations:** 1 Saga University, Saga City, Japan

## Background

When the invasion of sepsis or trauma is applied to the organism, systemic inflammation occurs. In the cascade from invasion to inflammation, as one of the chemical mediators of systemic inflammation, the relationship of HMGB1 to the onset of AKI and ARDS has been pointed out. In this study we investigated the ability to remove HMGB1 by the hemofiltration membrane *in vitro*.

## Methods

We dissolved HMGB1 in bovine serum, using a blood filtration membrane filter made of two types of membrane: heparin graft AN69ST (oXiris) and a membrane filter made of polyarylether sulfone (HF set). Filtration experiments were performed *in vitro*. The HMGB1 concentrations of the serum of inlet and outlet of the filter and of the filtrate were measured over time up to 120 minutes. Using an electron microscope, HMGB1 adsorption on the membrane was taken.

## Results

The HMGB1 concentration (ng/ml) immediately after and 15 minutes after the start was oXiris: 15.4 ± 1.9 and 8.6 ± 1.2, HF set: 16.0 ± 0.8 and 16.5 ± 1.1 (mean ± SD). The concentration was significantly decreased in 15 minutes immediately after the start of the oXiris set. The blood clearance (ml/minute) immediately after the start was oXiris: 44.6 ± 2.51, HF set: 7.77 ± 2.35. The filtrate clearance was oXiris: 0.13 ± 0.23, HF set: 0.43 ± 0.21. In the electron microscope, HMGB1 was adsorbed on the membrane.

## Conclusion

oXiris removed HMGB1 in a short time; its mechanism has been shown to be adsorption. HMGB1 can be removed from the blood, and there is a possibility to control the excessive inflammatory response during severe invasion to an organism *in vivo*.

